# Bacteriocin-Producing Probiotic Lactic Acid Bacteria in Controlling Dysbiosis of the Gut Microbiota

**DOI:** 10.3389/fcimb.2022.851140

**Published:** 2022-05-16

**Authors:** Anjana, Santosh Kumar Tiwari

**Affiliations:** Department of Genetics, Maharshi Dayanand University, Rohtak, India

**Keywords:** probiotics, bacteriocins, dysbiosis, gut microbiota, modulation, immunity

## Abstract

Several strains of lactic acid bacteria are potent probiotics and can cure a variety of diseases using different modes of actions. These bacteria produce antimicrobial peptides, bacteriocins, which inhibit or kill generally closely related bacterial strains and other pathogenic bacteria such as *Listeria, Clostridium*, and *Salmonella*. Bacteriocins are cationic peptides that kill the target cells by pore formation and the dissipation of cytosolic contents, leading to cell death. Bacteriocins are also known to modulate native microbiota and host immunity, affecting several health-promoting functions of the host. In this review, we have discussed the ability of bacteriocin-producing probiotic lactic acid bacteria in the modulation of gut microbiota correcting dysbiosis and treatment/maintenance of a few important human disorders such as chronic infections, inflammatory bowel diseases, obesity, and cancer.

## Introduction

Gut microbiota is crucial in maintaining the host defense system and homoeostasis, protecting against pathogens, and strengthening the gut integrity. The majority of gut bacteria belong to Bacteroidetes (e.g., *Porphyromonas, Prevotella*), Firmicutes (e.g., *Enterococcus*, *Lactobacillus*, *Streptococcus*, *Ruminococcus, Clostridium*), Actinobacteria (e.g., *Bifidobacteria*), and Proteobacteria (e.g., *Escherichia coli*) (Zhang et al., 2015). The perturbation in the commensal gut microbial flora causes dysbiosis, which happens due to several factors like imbalanced diet, infection, or the use of antibiotics, which can cause a long-term shift in the gut commensal microflora, promoting a large number of deadly diseases (Lange et al., 2016). There are several diseases associated with the dysbiosis of intestinal microbiota such as viral infections, inflammatory bowel disease (IBD), Crohn’s disease (CD), colorectal cancer, and obesity (Kim et al., 2019). Dysbiosis results in the development of diseases related to immune deregulation such as allergy and autoimmune and inflammatory disorders (D’amelio and Sassi, 2017). The colonization of gut by bacteriocin-producing probiotic strains inhibits the adhesion of pathogen to intestinal epithelial cells through competition, clearing niche, and spatial segregation (Heilbronner et al., 2021). Probiotics are living microorganisms that, upon ingestion in an adequate amount, provides a health benefit to a host by improving the intestinal microflora (Binda et al., 2020). Bacteriocins are antimicrobial peptides produced by these bacteria that generally inhibit/kill pathogenic bacteria in the gut and change the composition of gut microbiota in animal models such as mice, pigs, and chickens (Gillor et al., 2008; Yang et al., 2014). The mode of action of bacteriocins is different from antibiotics, which kill the target cells by pore formation and membrane disruption. Moreover, bacteriocins, being ribosomally synthesized proteins, are degraded by proteolytic enzymes, and therefore, the pathogens are not able to develop resistance in the gut (Epand et al., 2016; Umu et al., 2017). Further, bacteriocins have a simpler biosynthetic mechanism and are easy to increase their activity against target microorganisms with the help of bioengineering as compared to conventional antibiotics. In addition, a higher specific activity against multidrug-resistant pathogens offers advantages for their applications in therapeutics (Perez et al., 2014). Therefore, the use of bacteriocins and/or bacteriocin-producing probiotics is a novel approach for the treatment of several diseases including enteric infections and the restoration of health-promoting microbial community (Fong et al., 2020).

## Lactic Acid Bacteria

Probiotic lactic acid bacteria (LAB) are a nonpathogenic heterogeneous group of catalase-negative, Gram-positive, non-sporulating bacteria. They produce lactic acid as a main product from glucose and several growth-inhibiting substances like bacteriocins, bacteriocin-like inhibitory substances (BLISs), hydrogen peroxide, diacetyls, and carbon dioxide. These bacteria need complex nutritional substances for growth such as amino acids, peptides, nucleotide bases, vitamins, fatty acids, and carbohydrates (Mokoena, 2017). They are found in dairy products, fermented meats, fishes, beverages, pickled vegetables, and cereals and in the cavities of human and animals. Important genera include *Lactococcus, Enterococcus, Streptococcus, Pediococcus, Aerococcus, Alliococcus, Carnobacterium, Dolosigranulum, Oenococcus, Tetragenococcus, Vagococcus, Weissella*, and *Lactobacillu*s being the largest genus (Bintsis, 2018).

Lactobacilli are the most common probiotics found in humans and other animals. The major species of lactobacilli found in the gut are *L. gasseri*, *L. crispatus, Limnosilactobacillus reuteri, Ligilactobacillus salivarius*, and *L. ruminis* (Walter, 2008; Zheng et al., 2020). A metagenomic analysis suggests that therapy using a combination of different species of *Bifidobacterium* and *Lactobacillus* remarkably changes the composition of intestine microbiota in mice (Azad et al., 2018). Other than LAB, *Bifidobacterium* is considered as the first gut-colonizing microbe that exerts health benefits to the host (O’Callaghan and Sinderen, 2016). In breastfeeding infants, the species of *Bifidobacterium* are present in a wide range that gradually change with age. *B. longum, B. bifidum*, and *B. breve* are generally dominant in the gut of infants, whereas *B. catenulatum, B. adolescents*, and *B. longum* are present in adults. They may be used as remedy for the treatment/maintenance of various gastrointestinal (GI) diseases and restrict the deleterious microorganisms, enhance the GI fence, and inhibit proinflammatory cytokines (Xue et al., 2017).

Bacteria other than LAB also dominate the gut and play crucial functions. For example, harmless *E. coli* Nissle, found in the gut, is a widely utilized probiotics used for the balance of intestine microbiota. It has been revealed that it can restore the production of human β-defensin 2 that could save an intestinal hurdle in opposition to the adherence and capture by pathogenic *E. coli* (Schlee et al., 2007; Liu et al., 2017). Thus, LAB are a major component of gut microbiota that play an important role in maintaining the balance of the total microbial community.

## Bacteriocins

Bacteriocins are multifunctional, antimicrobial peptides produced by different bacteria that act at low concentrations and generally inhibit the growth of closely related species (narrow spectrum), but recent findings also suggest the occurrence of broad-spectrum bacteriocins (Chi and Holo, 2018; Goyal et al., 2018). Bacteriocin-producing cells are resistant to these antimicrobial peptides due to the presence of immunity proteins on the cell membrane of the producer bacteria. Nisin, a bacteriocin produced by several strains of *Lactococcus lactis*, has received GRAS (generally regarded as safe) status by the American Food and Drug Administration (FDA) and is generally used in food safety (Negash and Tsehai, 2020). According to Zacharof and Lovitt (2012), bacteriocins have been classified into three classes on the basis of biochemical and genetic characteristics: class I bacteriocins are lantibiotics with molecular weight < 5 kDa; they are posttranslationally modified, leading to the formation of methyllanthionine and lanthionine. Class II bacteriocins are non-lantibiotics with molecular weight <10 kDa, non-modified, heat stable, and further divided into three subclasses: class IIa peptide with anti-listerial activities such as pediocinPA1/AcH from *Pediococcus* species contain the N-terminal conserve sequence YGNGVXC (Nishei et al., 2012); class IIb consists of two peptide bacteriocins such as lactococcin G from *L. lactis*; in Class IIc, N- and C-terminals are linked by peptide bond forming cyclic bacteriocins, e.g., enterocin AS-48 (Van Belkum et al., 2011). Class III consists of heat-stable and large-sized bacteriocins with molecular weight > 30 kDa, e.g., enterolysin and helviticin (Yang et al., 2014).

Bacteriocins are effective against various human infectious diseases due to their efficacy against several pathogens. For example, pneumonia, meningitis, and sepsis caused by *Streptococcus pneumonia* can be treated with nisin (Goldstein et al., 1998). The cyclic bacteriocin griselimycin was able to cure tuberculosis in mice (Kling et al., 2015). It has been reported that a few bacteriocins such as pediocin PA-1 and lactocin AL705 show anticancer and anti-inflammatory activities (Huang et al., 2021). For example, nisin inhibits the proliferation of cancer cells by the formation of ion channels on the cell membrane, releasing lactate dehydrogenase, increasing the number of reactive oxygen species, and obstructing the mitochondrial respiration of cancer cells. It was also reported that nisin, in combination with cancer drugs, shows synergistic activity in the clearance of a tumor (Preet et al., 2015). Bacteriocins increase the anti-inflammatory cytokine level and decrease the pro-inflammatory cytokine level by various signaling pathways such as mitogen-activated protein kinase and Toll-like receptor (Sassone-Corsi et al., 2016). Thus, bacteriocins are important probiotic metabolites of LAB that can be used for different health-promoting activities of the host.

## Bacteriocin-Producing Lactic Acid Bacteria

There are sufficient studies describing the effect of bacteriocin-producing LAB on changing gut microbiota in animals and humans (Yang et al., 2014; Hernandez-Gonzalez et al., 2021). For example, bacteriocin Abp118, produced by *L. salivarius* UCC118 isolated from the terminal ileum of a human intestine, shows antilisterial activity in the gut of murine and porcine (Riboult-Bisson et al., 2012). There is change in fecal bacteria community in humans caused by *L. plantarum* P-8, which is due to the production of plantaricin (Kwok et al., 2015). In another study, intraperitoneally injected nisin F produced by *L. lactis* ssp. *lactis* F10 showed a stabilizing effect on bacterial community in the gut of mice (Umu et al., 2017). Another bacteriocin, thuricin CD composed of two peptides, Trnα and Trnβ, is secreted by *Bacillus thuringiensis* DPC6431, which kills a wide range of *C. difficile* isolates without affecting commensal microbiota in a distal colon model (Rea et al., 2010).

Bacteriocin-producing LAB are effective against infections caused by foodborne pathogens like *Listeria monocytogenes* and several enterococci present in human intestine (Harris et al., 1989; Millete et al., 2008). *Pediococcus acidilactici* UL5 produces pediocin PA-1, which showed anti-listerial activity in a mouse model without affecting the native intestinal microflora (Dabour et al., 2009). The administration of enterocin CRL35 produced by *E. mundtii* CRL35 in pregnant mice inhibited the transfer of *L. monocytogenes* to vital organs (Salvucci et al., 2012). Plantaricin PJ4 produced by *L. helveticus* PJ4 isolated from the gut of rat showed potent results in reducing weight in obese mice (Bai et al., 2020). Similarly, plantaricin EF produced by *L. plantarum* NCMIB8826 shows a beneficial effect in diet- induced obese mice (Heeney et al., 2019). The immunomodulatory and immunostimulatory effects of nisin (in the form of commercial preparation)-containing diet was evaluated and increased the CD4 and CD8 T lymphocytes and reduced the B-lymphocyte cell count (Pablo et al., 1999). In another *in vivo* study, a reduction in the colonization of vancomycin-resistant enterococci (VRE) in the intestine of the mice model was reported by administering the bacteriocin producer, *L. lactis* MM19 and *P. acidilactici* MM33 isolated from the fecal sample of a human (Millete et al., 2008). When rats were administered *S. aureus* K followed by treatment with nisin F intranasally, immunosuppressed rats showed pneumonia symptoms that had not been administered with nisin F, while the rats colonized by *S. aureus* K and treated with nisin F showed a healthy trachea and lungs (De Kwaadsteniet et al., 2009).

Umu et al. (2016) demonstrated the effect of bacteriocin-producing LAB and their isogenic mutants on the modulation of gut microbiota. The bacteriocins used in this study were sakacin A, pediocin PA-1, enterocin P, Q, and L50. They demonstrated that the oral administration of a bacteriocin producer does not change the overall structure, but some beneficial changes occur at a lower taxonomic level in the mice gut, whereas some changes were reversed back after treatment. It was interesting to know that the isogenic mutant of respective strains did not cause such changes, suggesting the role of bacteriocin in the modulation of microbiota (Umu et al., 2016). Oral administration of probiotics such as *Lacticaseibacillus casei, L. acidophilus, L. plantarum*, and *Streptococcus thermophiles* in a dose-dependent manner enhanced the number of Ig-A- and Ig-G-producing cells (Azad et al., 2018). The administration of nisin Z and pediocin AcH reduced the colonization of the pathogen when given 8 days prior to infection with vancomycin-resistant *Enterococcus* (Millette et al., 2008). There is alteration in the composition of gut microbiota when administered with a combination of probiotics, e.g., *L*. *ramnosus, L. acidophilus*, and *B. bifidum*, in mice fed with a high-fat diet (Azad et al., 2018). Thus, there is enough evidence suggesting the role of bacteriocin-producing probiotic LAB in modulating gut microbiota and maintaining host health. Bacteriocin-producing LAB used for the treatment of several diseases are mentioned in **Table 1**.

**Table 1:** Bacteriocin-producing lactic acid bacteria involved in the modulation of gut microbiota and treatment/maintenance of different diseases.

S. No.	Lactic acid bacteria	Bacteriocins	Diseases/target pathogens	Model	References
1	*Lactococcus lactis* DPC3147	Lacticin3147,	*Clostridium difficile* associated diarrhea (CDAD)	*in vitro*	(Rea et al., 2007)
2	*L. garvieae*	Garvicin ML	Active *Streptococcus pneumonia*.	*in vitro*	(Borrero et al., 2011)
3	*L. lactis*	Nisin Z	Immunomodulatory effect	Murine model	(Millette et al., 2008)
4	*L. lactis*	Nisin F	Respiratory infection	Murine model	(De Kwaadsteniet et al., 2009)
5	*L. lactis*	Nisin	Meniningitis, sepsis, pneumonia	*in vitro*	(Goldstein et al., 1998)
6	*L. lactis*	Nisin Z	Enteric pathogens	Mouse model	(Millette et al., 2008)
7	*L. lactis*	Nisin A	Colorectal cancer	*in vitro*	(Norouzi et al., 2018)
8	*L. lactis*	Nisin	Stress reduction	Mice model	(Jia et al., 2018)
9	*Lactobacillus salivarius*	Bacteriocin Abp118	Listeriosis	Murine model	(Riboult-Bisson et al., 2012)
10	*L. salivarius* NRRLB	Bacteriocin OR-7	*Campylobacter jejuni*	Chicken model	(Ilinskaya et al., 2017)
11	*L. salivarius*	Bactofencin A	Antilisterial, antistaphylococcal	*in vitro*	(O’Conner et al., 2018)
12	*L. curvatus*	Lactocin AL705	Listeriosis	*in vitro*	(Huang et al., 2021)
13	*L. rhamnosus *	Lactocin 160	*Escherichia coli, Bordetella pertussis*	*in vitro*	(Belfiore et al., 2007)
14	*Pediococcus acidilactici*	Pediocin PA1	Listeriosis	Murine model	(Dabour et al., 2009)
15	*P. acidilactici*	Pediocin AcH	Enteric pathogens	Mouse model	(Millette et al., 2008)
16	*P. acidilactici*	Pediocin	Colorectal cancer	*in vitro*	(Kaur and Kaur, 2015)
17	*P. acidilactici* K2a2-3	Pediocin PA-1	Anti-cancerActivity	*in vitro*	(Huang et al., 2021)
18	*Enterococcus mundtii* RL35	Enterocin CRL35	Listeriosis	Murine model	(Salvucci et al., 2012)
19	*E. avium*	Avicin	Listeriosis	*in vitro*	(Birri et al., 2010)
20	*E. faecium* P13	Enterocin P	Enteric pathogens	*in vitro*	(De Kwaadsteniet et al., 2006)
21	*E. mundtii* RL35	Enterocin CRL35	Herpes virus	*in vitro*	(Wachsman et al., 1999)
22	*E. faecium* ST5Ha	Bacteriocin ST5Ha	Herpes virus	*in vitro*	(Todorov et al., 2010)
23	*Carnobacterium maltaromaticum*	Piscicolin 126, carnobacteriocin	Listeriosis	Pork model	(Martin-Visscher et al., 2008)
24	*Streptomyces* spp.	Griselimycin	*M. tuberculosis*	*in vivo*	(Kling et al., 2015)
25	*Leuconostoc citreum* GJ7	Kimchichin	*Salmonella typhi*	*in vitro*	(Chang and Chang, 2011)
26	*Erwinia carotovora* NA4	Erwinaocin NA4	Coliphage	*in vitro*	(Dey et al., 2021)

## Gut Microbiota and Immune Modulation

The mucosal immune system protects GI tract from evading pathogens. Mucosa associated lymphoid tissue, epithelial layer and lamina propria are the main parts of the immune system. *L. fermentum, L. crispatus*, and *L. gasseri* are known to interact with dendritic, enterocytes and Treg cells in human GI tract and adaptive immunity is activated to release pro- and anti-inflammatory cytokines (Azad et al., 2018). There is modulation in an innate and adaptive immune system by the antigenic fragments of probiotic strains as they are capable to enter into the intestinal epithelial cells and M cells of Peyer’s patches. Cytokines such as interleukin (IL), tumor necrosis factor (TNF), and interferon (IFN) regulate the innate immune system. Similarly, differentiation of CD8+ T-lymphocyte cells into cytotoxic T-lymphocytes kills the virus-infected cells and activates natural killer cells and macrophages, destroying pathogens (Singh and Rao, 2021).

It was reported that fermentation products of the probiotic *Bifidobacterium breve* C-50 trigger the maturation of dendritic cells and promote the survival of dendritic cells (DCs) and IL-10, which show an anti-inflammatory response. Prolonged survival of DC is caused by increased levels of antiapoptotic protein; BCL-xl triggers PBK/Akt phosphorylation, causing maturation by elevating the effect of CD86 and CD83 maturation markers (Hoaru et al., 2006). DC protects feasible gut microflora and dispatches microorganisms to “mesenteric lymph nodes” and results in the production of IgA antibodies to defend the opposition of mucosal invasion (Macpherson and Uhr, 2004; Macpherson et al., 2005). The differentiation of naive T cells into different types of cell lines like TH-17, TH-2, TH-1, CD8+ repressor, and regulatory T cell depends upon the interaction of DC with specific pattern recognition factors. A study states that cytophage*-bacteroides* required for development of TH17 cells in lamina propria, which, in turn, maintains the balance between regulatory T-cell populations and TH-17 (Foligne et al., 2007; Delcenserie et al., 2008; Zeuthen et al., 2008). Evidence proved that when germ-free mice were colonized with *Bacteroides fragilis* NCTC9343, an immense restoration was observed in the number of CD4+ CD45Rb T-cell populations. This restoration was caused by *B. fragilis* polysaccharide A (Mazmanian et al., 2008). Interestingly, it was observed that mutant strain lacking this polysaccharide A failed to restore the number of CD+ CD45Rb T-cell populations. In a model of colitis, when *L. paracasei* was administered intragastrically, it rendered a protective effect by reducing the severity of diseases and delaying their progression (Mileti et al., 2009). It was observed that naïve T cells acquired suppressor functions when DC was treated with any one of these probiotics: *Streptococcus thermophilus* DN-001 621, *Bacteroides adolescentesis* DN-150 017, and *Bifidobacterium animalis* DN173 016. The suppressive effect was caused by the decrease in the proliferation of differentiated T cells and IFNγ production by CD4+ effector T cells (Baba et al., 2008). Nisin showed an immunomodulatory effect in the mice model, resulting in an increase in CD4+ and CD8 T lymphocytes, with decreased B lymphocytes and its administration for a long duration might balance the level of B and T lymphocytes. Nisin Z was effective in modulating the innate immune response by lowering the level of proinflammatory cytokines in human peripheral blood mononuclear cells (PBNCs). It can be used in periodontal disease in which there is an initial burst of neutrophils and in its later stages, B- and T-cell-related immune response was shown by the immune system (Shin et al., 2016).

## Gut Microbiota and Gut–Brain Axis

Gut microbes generally secrete amino acids that interact with the ganglion cells (Dinan and Cryan, 2017) in response to the central nervous system (CNS) pattern of chemical messengers (Sanders et al., 2011; Briguglio et al., 2018). Various passages of interaction in the middle of the intestine and CNS have been studied (Dinan and Cryan, 2017). The vagus nerve performs a relationship in the middle of the intestine and spinal cord, which is terminated in the brain stem nuclei and is tactile to deviating fibers (Bonaz et al,. 2018),. Thus, the brain stem nuclei may influence numerous bowel roles and convey gestures to more CNS zones, such as the midbrain along with the cerebral cortex (Wang and Wang, 2016). An interchange in the middle of intestine and CNS can also take place through systemic blood flow (Gibson and Mehler, 2019).

The microbiota–gut–brain axis can be noticed as a web with various functions, where midway and sideways, the immune and endocrine systems take part into duplex transmission (Borre et al., 2014). Initially, microbes are capable to substitute, combine, and break neurotransmitters along with transmodulators, like acetate, propionate, butyrate histamine, other pyrimidines, and glutathione (Dinan and Cryan, 2017). These substances act as a neurotransmitter in the brain and stabilize the neuronic venture. However, there is a need of a detailed study to show the direct effect (Angelucci et al., 2019). Furthermore, the gut microbiota produce other proteins that are the deleterious substitute of CNS, being robust irritant cytokines and inborn response activator B cells in the host (Alam et al., 2017). Thus, native microbiota can influence the microbiota–gut–brain axis through antibody-mediated nervous and endocrine systems along with various pathways (Gibson and Mehler, 2019). The outcome to these neural changes in the brain can guide to destruction, hypertension, and other coherent diseases (Saunders et al., 2002; Galland, 2014; Johnson and Foster, 2018; Angelucci et al., 2019). Alteration in the gut microbiota is connected to several neurological disorders (Cox and Weiner, 2018), which involve not only hypertension and stress (Nagpal et al., 2018) but also neurodegenerative diseases (Quigley, 2017) and refractory epilepsy (Braakman and Van Ingen, 2018).

Till date, there is no direct evidence available suggesting the role of bacteriocins or bacteriocin-producing LAB in the gut–brain axis. However, gut microbiota may be modulated with producer strains and may indirectly influence the gut–brain axis. In an *in silico* study, the beneficial effects of nisin on neurotransmitter, aquaporin, and commensal gut microbiota were analyzed using high-throughput sequencing, which provided the relationship between the gut microbiota and the neurochemicals used in the gut–brain axis. Nisin showed the highest expression of norepinephrine in the brain as compared to the control group and ciprofloxacin-treated group. Further, it was found that mice treated with nisin showed an increased level of *Lactobacillus*, Bacteroides, and *Bifidobacterium* and decrease in pathogenic *E. coli* and enterococci in the cecum sample. Thus, there is a strong relation between nisin, gut bacterial flora, and reduction in stress triggered by *E. coli* in the mice model (Jia et al., 2018).

## Bacteriocin-Producing Lactic Acid Bacteria and Their Role in Diseases

**Figure 1:**
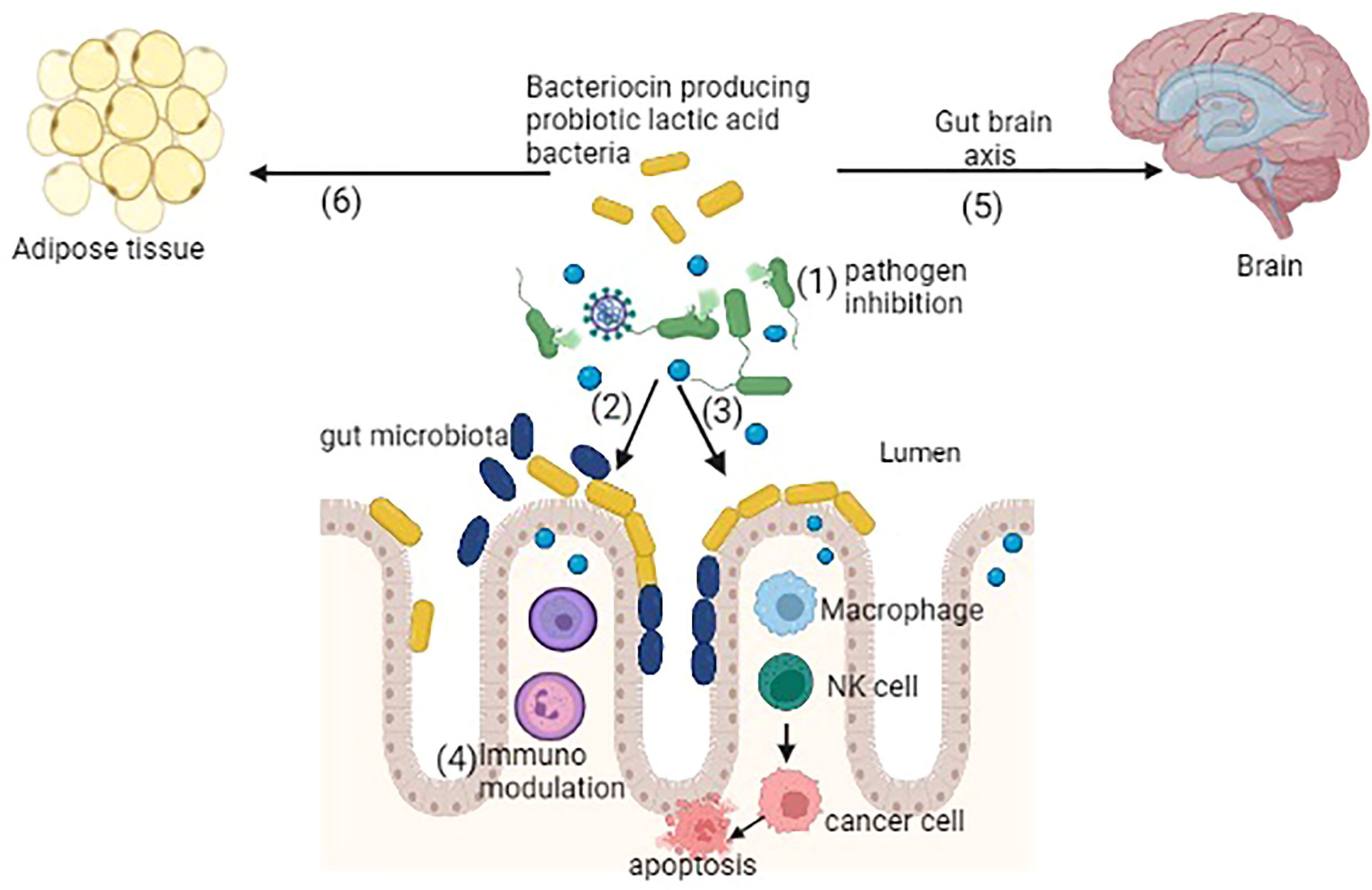
Bacteriocin-producing probiotic lactic acid bacteria showing several potential functions: (1) inhibition of pathogens, (2) colonization of probiotic bacteria by competitive exclusion, (3) activation of macrophages, natural killer (NK) cells further interact with cancer cells causing apoptosis, (4) immunomodulation, (5) gut–brain axis balancing the gut microbiota, (6) antiobesity activity by reducing the adipose tissue (created in BioRender).

Alteration in the normal microbiota of gut causes several chronic diseases, like joint pain, immune-related diseases, metabolic disorders, liver diseases, and various GI diseases (Carding et al., 2015). Bacteriocins may play a role in shaping the host microbiota and indirectly play an important role in correcting dysbiosis and the improvement of host health. Here, we have discussed a few important diseases that occur during the dysbiosis of the gut and their possible cure using bacteriocin-producing probiotic lactic acid bacteria. For clarity, a diagrammatic presentation of the same is depicted in **Figure 1**.

### Colonic Infections

Bacteriocins and/or the bacteriocin-producing strains of LAB are documented for the inhibition of several foodborne and clinical pathogens causing severe infections. Most of the LAB bacteriocins are pore formers and interact with the cell membrane to kill the target bacteria through the dissipation of membrane potential and ATP efflux, leading to cell death. Thus, the bacteriocins of LAB may serve as an alternative to clinical antibiotics and can be applied to treat bacterial infections (Pérez-Ramos et al., 2021; Sheoran and Tiwari, 2021; Li et al., 2022).

*Clostridium difficile* is the main causative bacterium for colonic infection. Using an *ex vivo* model, it was found that purified nisin was selectively able to deplete *C. difficile* in a fecal microbial environment without affecting native gut microbiota. The other pathogenic bacteria are *E. coli, Salmonella typhi, Campylobacter jejuni, Shigella*, and *Yersinia enterocolitica* (Papaconstantinou and Thomas, 2007). Such infections were found to be minimized by increasing the population of commensal probiotic *L. acidophilus* (Yun et al., 2014). The combination of lactocin and a chelating agent, ethylenediamine tetraacetic acid, was found effective against *E. coli* (Belfiore et al., 2007). *L. salivarius* NRRLB produces the bacteriocin OR-7, which is active against an enteric pathogen, *C. jejuni*, in the human GI tract (Ilinskaya et al., 2017). Enterocin P inhibits *Staphylococcus, Clostridium, L. monocytogenes, Enterococcus faecium*, and *E. faecalis* (De Kwaadsteniet et al., 2006). The inhibitory effect of the bacteriocin-producer *L. casei* against *E*. *coli* and *L. monocytogenes* was found in the mice model (Soltani et al., 2021). Kimchicin GJ7 produced by *L. citreum* GJ7 inhibited *S. typhi in vitro* (Chang and Chang, 2011). Bacteriocin BM1829 produced by *L. crustorum* MN047 inhibited *E. coli, S. typhi*, and *S. aureus* by arresting the cell cycle at the G_1_/S checkpoint or destructing the membrane integrity (Yan et al., 2021).

During the ongoing pandemic, viral infections have caused severe diseases and mortality. There are many antiviral agents proposed and tested that have recently been proven successful in treating such infections. However, these therapies showed toxicity and were not able to reduce the symptoms completely. Therefore, it is important to find a safe alternative for the treatment of viral infections (Lehtoranta et al., 2020; Villena et al., 2020). Probiotic LAB exert their antiviral effect by direct interaction with viruses, producing bacteriocins, or enhancing the innate immunity of the host (Al Kassaa et al., 2014; Tiwari et al., 2020). Bacteriocins show antiviral activity against a number of viruses by blocking the synthesis of glycoprotein in the late stage of virus replication (Huang et al., 2021). Acute gastroenteritis is mainly caused by rotavirus, norovirus, and adenovirus in children below 5 years of age. Rotavirus is a double-stranded RNA-lacking envelope that causes the destruction of epithelial cell lining in infants, causing diarrhea (Li et al., 2021). *Lacticaseibacillus rhamnosus* GG suppresses human rotavirus and induced autophagy in the intestine of piglets by lowering the amount of autophagy proteins, p-mTor, and VPS34-positive cells, Beclin 1 and ATG16L1. *L. rhamnosus* GG also increases the level of p53 proteins and induces the apoptosis of infected intestinal cells (Wu et al., 2013).

A few enzymatic reactions important for viral infection are inhibited by bacteriocin or bacteriocin-like substances (Salman et al., 2020). It was observed that oral administration of nisin increases the level of CD4+ and CD8+ T lymphocytes and reduces the B cells in mice (Dey et al., 2021). Enterocin CRL35 produced by *Enterococcus faecium* CRL35 inhibits the replication of the herpes simplex virus, which causes gut-related ulcerative diseases in humans (Wachsman et al., 1999). Erwiniaocin NA4 produced by *Erwinia carotovora* NA4 kills the coliphage HSA, and enterocin NKR-5-3C produced by *Enterococcus faecium* NKR-5-3 shows antagonistic activity against HSV type 1 (Dey et al., 2021). Nisin and sakacin A are effective against the non-enveloped murine norovirus, and bacteriocin ST5Ha produced by *Enterococcus faecium* ST5Ha shows antiviral activity against the herpes simplex virus (Todorov et al., 2010; Lange-stark et al., 2014). Norovirus is an enteric virus with non-enveloped, single-stranded RNA, which belongs to the family Calciviridae. There is an increase in the number of proteobacteria and a decrease in Bacteroidetes in a norovirus-infected person. The direct binding of norovirus to fecal isolated proteobacteria indicates the modulation of gut microbiota. Further, the attachment of P-particles present on the virus to epithelial cells can be inhibited by *L. casei* BL23 (Salman et al., 2020). This evidence suggests that bacteriocins and/or the bacteriocin-producing strains of LAB have a potential in preventing the viral infection (Cavicchioli et al., 2018) and therefore, further research is essential to find out the exact mechanism of action before application in therapeutics.

### Inflammatory Bowel Disease

IBD is a long-term erythrogenic disease of the gastrointestinal tract that comprises ulcerative colitis (UC) and CD (Bjarnason et al., 2019). The etiology of IBD is not determined yet. It is generally aggravated by improper diet, the disruption of microbiota, and depression. However, a few intestinal microorganisms such as *E. coli, C. concisus*, and *Mycobacterium avium* are also involved in the pathophysiology of the IBD (Ryma et al., 2021). UC and CD illnesses have constant provocative states of etiology with various components including genetic susceptibility, hereditary inclination, ecological triggers, changes in the immune system, and an unusual response of gut microbiota. In these diseases, microbial imbalance occurs in a patient, which is characterized by dysbiosis (Sidhu and Vander Poorten, 2017). Fecal microbiota transplant is a potential treatment for IBD, but its success rate is low (Colman and Rubin, 2014). The efficacy of *Lactobacillus* GG as an adjuvant has been studied in maintaining the remission in CD patients (Scaldaferri et al., 2013). Probiotics seem to be effective and well tolerated by IBD patients, but the role of bacteriocin and its mechanism is still unknown (Ryma et al., 2021).

The microbiota of patients related to IBD is distinct from healthy individuals (Shadnoush et al., 2015). It was observed that the number of Firmicutes like *Faecalibacterium prausnitzi*, which is one of the most abundant gut bacteria, and Bacteroidetes decreased and Proteobacteria and Actinobacteria were increased during IBD. Thus, it is necessary to stabilize the gut microbiota to overcome such diseases where bacteriocin-producing probiotics can play a significant role by promoting the growth of healthy microbiota (Furrie et al., 2005), although a study has shown that probiotic supplementation in IBD is favorable for the cure of ulcerative colitis but not CD (Bjarnason et al., 2019). Alterations in the gut microbiota cause a defect in the mucus layer that increases the intestinal permeability to pathogens and triggers an immune response, causing intestinal inflammation (Michielan, and D’Incà, 2015). Bacteriocin can maintain the integrity of gut epithelium by directly inhibiting/killing the pathogen or can act as a colonizing peptide promoting LAB to occupy niches in the intestine. *L. reuteri* is a commensal bacterium of gut secretion reuterin, which inhibits several enteropathogens such as yeast, fungi, protozoa, and viruses and promotes the growth of beneficial Gram-positive bacteria (Liu et al., 2020). It was found that probiotics and their metabolites such as short-chain fatty acids play an important role in intestinal dysbiosis and the immunopathogenesis of IBD (Ryma et al., 2021). In a recent study, it was reported that the bacteriocin-producing strains of *L. casei, L. plantarum, L. rhamnosus*, and *L. acidophilus* isolated from breast milk competed with intestinal pathogens, reduced the human colorectal adenocarcinoma cell line (HT-29), lowered cholesterol levels, and improved IBD in the mice model (Abdi et al., 2021).

### Colorectal Cancer

Colorectal cancer affects the rectum and colon of the large intestine with major symptoms of bloody stool and reduced body weight. It depends on various factors such as diet, lifestyle, and aging (Center et al., 2009). The efficacy of potent LAB was demonstrated through prominent clinical investigation and animal model experiments (Krebs, 2016). The study on the mice model provides evidence that *Bifidobacterium* with bacteriocin-producing probiotic combination reduces the chances of colorectal cancer (O’Callaghan and Sinderen, 2016). *L. acidophilus* alone or in combination was found to boost the immunity against colorectal cancer (Zhong et al., 2014). Probiotic bacteria secrete numerous substances with anticancer activity including bacteriocins, toxins, and enzymes. Nisin A produced by *L. lactis* inhibits tumor cell growth and changes the membrane integrity of liver hepatocellular carcinoma (HepG2). Nisin forms pores in the cell membrane and induces apoptosis through an intrinsic pathway and also acts as an antimetastatic agent by lowering the proliferation of melanoma cells (Norouzi et al., 2018). In addition, pediocin produced by *P. acidolactici* K2a2-3 inhibits the proliferation of human colon adenocarcinoma cells (HT29) (Soleimanpour et al., 2020).

The *in vitro* effect of colicin E7 produced by *E. coli* on the HT-29 cell line was evaluated for the expression of p53, and bcl-2 shows a decrease in bcl-2 and increase in p53 gene expression (Taherikalani and Ghafourian, 2021). Microcin causes cell membrane depolarization, the fragmentation of DNA, release of phosphatidylserine, and caspase activity (Baindara et al., 2018). In an *in vitro* study, pediocin produced by *Pediococcus acidilactici* K2a2-3 shows an anticancer activity on HT-29 and DLD-1 cell lines in a dose-dependent manner (Kaur and Kaur, 2015). This evidence suggests the role of bacteriocin either directly or indirectly for the cure of colorectal cancer.

### Obesity

Obesity is a metabolic disorder closely related to dysbiosis in the gut microbiota. Probiotics are helpful in modulating the gut microbiota to combat such disorders. The gut microbiota are involved in balancing energy intake and satiety through gut peptide signaling or altering the nervous system. The balance of the regulatory signaling peptide is altered if there is a change in gut microbiota. Hence, obesity can be cured by restoring the gut microbiota. There is change in the ratio of Firmicutes/Bacteroidetes in obese people (Mazloom et al., 2019). The imbalance was identified by a decrease in the number of Gram-negative aerobes and anaerobe Bacteroidetes and increase in Gram-positive Firmicutes (Sze and Schloss, 2016). However, weight gain and fit metabolic physiology in mice could be passed on *via* fecal/stool microbiota transplant (Turnbaugh et al., 2008; Liou et al., 2013). Bacteriocin-producing probiotics decrease the absorption of fatty acids and reduce the size of adipocytes and also increases the expression of genes related to oxidation of fatty acids (Wicinski et al., 2020). *L. plantarum* stimulates the production of TNFα and also regulates the production of leptin hormones (Behrouz et al., 2017).

Probiotics indirectly affect obesity by the production of bacteriocin, which modulates the bacterial content (Million et al., 2013). Treatment with *L. mali* APS isolated from kefir reduces obesity in the mice model. Bacteriocin PJ4 produced by *L. helveticus* is proven to be effective in reducing the inflammation and body weight in the mice model (Bai et al., 2020). Heeney et al. (2019) investigated those mice fed with plantaricin EF-producing *L. plantarum* NCMIB8826 reduced the consumption of high-fat diet and exhibited approximately 10% reduction in weight gain. The same was absent in the group supplemented with the isolgenic (Δ*plnEFI*) mutant strain LM0419.

## Conclusions and Future Perspective

The bacteriocins produced by probiotic lactic acid bacteria are generally small cationic peptides that kill the target cells by pore formation. These peptides show antimicrobial activity against related strains and pathogenic bacteria such as *Salmonella, Staphylococcus, Listeria, Clostridium*, and *Enterococcus*. Bacteriocins are also effective against viral infections caused by rotavirus, norovirus, adenoviruses, etc. Gut microbiota is an important part of human body and play a key role in stabilizing several body functions. Probiotics and their bacteriocins have potential in modulating the gut microbiota through antimicrobial action and immune modulation and are thus helpful in restoring the balanced microbial community in the gut and host immunity. In addition, the role of bacteriocins has also been demonstrated in colorectal cancer, IBD, and obesity. Thus, there is further need to characterize probiotic bacteria in the gut for their bacteriocin profiling and their role in the establishment of ecological niche of the gut using advanced techniques such as metagenomics, proteomics, and metabolomics. Such inventions will lead the discovery of nature-derived novel products and strategies for the cure of several chronic disorders.

## Author Contributions

All authors have made a substantial, direct, and intellectual contribution to the manuscript and approved it for publication.

## Conflict of Interest

The authors declare that the research was conducted in the absence of any commercial or financial relationships that could be construed as a potential conflict of interest.

## Publisher’s Note

All claims expressed in this article are solely those of the authors and do not necessarily represent those of their affiliated organizations, or those of the publisher, the editors and the reviewers. Any product that may be evaluated in this article, or claim that may be made by its manufacturer, is not guaranteed or endorsed by the publisher.

## References

[B1] Abdi M. Lohrasbi V. Asadi A. Esghaei M. Jazi F. M. Rohani M.. (2021). Interesting Probiotic Traits of Mother’s Milk Lactobacillus Isolates; From Bacteriocin to Inflammatory Bowel Disease Improvement. Microb. Pathog. 158, 104998. doi: 10.1016/j.micpath.2021.104998 34044041

[B2] Alam R. Abdolmaleky H. M. Zhou J. R. (2017). Microbiome, Inflammation, Epigenetic Alterations, and Mental Diseases. Am. J. Med. Genet. B: Neuropsychiatr. Genet. 174, 651–660. doi: 10.1002/ajmg.b.32567 28691768 PMC9586840

[B3] Al Kassaa I. Hober D. Hamze M. Chihib N. E. Drider D. (2014). Antiviral Potential of Lactic Acid Bacteria and Their Bacteriocins. Probiotics Antimicrob. Proteins 6, 177–185. doi: 10.1007/s12602-014-9162-6 24880436

[B4] Angelucci F. Cechova K. Amlerova J. Hort J. (2019). Antibiotics, Gut Microbiota, and Alzheimer’s Disease. J. Neuroinflamm. 16, 1–10. doi: 10.1186/s12974-019-1494-4 PMC6530014 31118068

[B5] Azad M. Kalam A. Sarker M. Li T. Yin J. (2018). Probiotic Species in the Modulation of Gut Microbiota: An Overview. Biomed. Res. Int. 2018, 1–9. doi: 10.1155/2018/9478630 PMC5964481 29854813

[B6] Baba N. Samson S. Bourdet-Sicard R. Rubio M. Sarfati M. (2008). Commensal Bacteria Trigger a Full Dendritic Cell Maturation Program That Promotes the Expansion of Non-Tr1 Suppressor T Cells. J. Leukoc. Biol. 84, 468–476. doi: 10.1189/jlb.0108017 18511576

[B7] Bai L. Kumar S. Verma S. Seshadri S. (2020). Bacteriocin PJ4 From Probiotic *Lactobacillus* Reduced Adipokine and Inflammasome in High Fat Diet Induced Obesity 3. Biotech 10, 1–10. doi: 10.1007/s13205-020-02317-y PMC7385047 32766096

[B8] Baindara P. Korpole S. Grover V. (2018). Bacteriocins: Perspective for the Development of Novel Anticancer Drugs. Appl. Microbiol. Biotechnol. 102, 10393–10408. doi: 10.1007/s00253-018-9420-8 30338356

[B9] Behrouz V. Jazayeri S. Aryaeian N. Zahedi M. J. Hosseini F. (2017). Effects of Probiotic and Prebiotic Supplementation on Leptin, Adiponectin, and Glycemic Parameters in Non-Alcoholic Fatty Liver Disease: A Randomized Clinical Trial. Middle East J. Dig. Dis. 9, 150. doi: 10.15171/mejdd.2017.66 28894517 PMC5585907

[B10] Belfiore C. Castellano P. Vignolo G. (2007). Reduction of *Escherichia Coli* Population Following Treatment With Bacteriocins From Lactic Acid Bacteria and Chelators. Food Microbiol. 24, 223–229. doi: 10.1016/j.fm.2006.05.006 17188201

[B11] Binda S. Hill C. Johansen E. Obis D. Pot B. Sanders M. E. (2020). Criteria to Qualify Microorganisms as “Probiotic” Foods Dietary Supplement. Front. Microbiol. 11, 1662. doi: 10.3389/fmicb.2020.01662 32793153 PMC7394020

[B12] Bintsis T. (2018). Lactic Acid Bacteria as Starter Cultures: An Update in Their Metabolism and Genetics. AIMS Microbiol. 4, 665–684. doi: 10.3934/microbiol.2018.4.665 31294241 PMC6613329

[B13] Birri D. J. Brede D. A. Forberg T. Holo H. Nes I. F. (2010). Molecular and Genetic Characterization of a Novel Bacteriocin Locus in *Enterococcus Avium* Isolates From Infants. Appl. Environ. Microbiol. 76, 483–492. doi: 10.1128/aem.01597-09 19933345 PMC2805230

[B14] Bjarnason I. Sission G. Hayee B. H. (2019). A Randomised, Double-Blind, Placebo-Controlled Trial of a Multi-Strain Probiotic in Patients With Asymptomatic Ulcerative Colitis and Crohn’s Disease. Inflammopharmacology 27, 465–473. doi: 10.1007/s10787-019-00595-4 31054010 PMC6554453

[B15] Bonaz B. Bazin T. Pellissier S. (2018). The Vagus Nerve at the Interface of the Microbiota-Gut-Brain Axis. Front. Neurosci. 12, 49. doi: 10.3389/fnins.2018.00049 29467611 PMC5808284

[B16] Borre Y. E. Moloney R. D. Clarke G. Dinan T. G. Cryan J. F. (2014). The Impact of Microbiota on Brain and Behavior: Mechanisms and Therapeutic Potential. Microbial Endocrinol.: Microbiota-Gut-Brain Axis Health Dis. 2014, 373–403. doi: 10.1007/978-1-4939-0897-4_17 24997043

[B17] Borrero J. Brede D. A. Skaugen M. Diep D. B. Herranz C. Nes I. F. (2011). Characterization of Garvicin ML, a Novel Circular Bacteriocin Produced by *Lactococcus Garvieae* DCC43, Isolated From Mallard Ducks (Anas Platyrhynchos). Appl. Environ. Microbiol. 77, 369–373. doi: 10.1128/AEM.01173-10 21057028 PMC3019728

[B18] Braakman H. M. Van Ingen J. (2018). Can Epilepsy be Treated by Antibiotics? J. Neurol. 265, 1934–1936. doi: 10.1007/s00415-018-8943-3 29931545

[B19] Briguglio M. Dell’Osso B. Panzica G. Malgaroli A. Banfi G. Zanaboni Dina C. (2018). Dietary Neurotransmitters: A Narrative Review on Current Knowledge. Nutrients 10, 1–15. doi: 10.3390/nu10050591 PMC5986471 29748506

[B20] Carding S. Verbeke K. Vipond D. T. Corfe B. M. Owen L. J. (2015). Dysbiosis of the Gut Microbiota in Disease. Microb. Ecol. Health Dis. 26, 1. doi: 10.3402/mehd.v26.26191 PMC4315779 25651997

[B21] Cavicchioli V. Q. de Carvalho O. V. de Paiva J. C. Todorov S. D. Júnior A. S. Nero L. A. (2018). Inhibition of Herpes Simplex Virus 1 (HSV-1) and Poliovirus (PV-1) by Bacteriocins From *Lactococcus Lactis* Subsp. Lactis and *Enterococcus Durans* Strains Isolated From Goat Milk. Int. J. Antimicrob. Agents 51, 33–37. doi: 10.1016/j.ijantimicag.2017.04.020 28668682

[B22] Center M. M. Jemal A. Smith R. A. Ward E. (2009). Worldwide Variations in Colorectal Cancer: CA Cancer . J. Clin. 59, 366–378. doi: 10.3322/caac.20038 19897840

[B23] Chang J. Y. Chang H. C. (2011). Growth Inhibition of Foodborne Pathogens by Kimchi Prepared With Bacteriocin-Producing Starter Culture. J. Food Sci. 76, M72–M78. doi: 10.1111/j.1750-3841.2010.01965.x 21535696

[B24] Chi H. Holo H. (2018). Synergistic Antimicrobial Activity Between the Broad Spectrum Bacteriocin Garvicin KS and Nisin, Farnesol and Polymyxin B Against Gram-Positive and Gram-Negative Bacteria. Curr. Microbiol. 75, 272. doi: 10.1007/s00284-017-1375-y 29058043 PMC5809525

[B25] Colman R. J. Rubin D. T. (2014). Fecal Microbiota Transplantation as Therapy for Inflammatory Bowel Disease: A Systematic Review and Meta-Analysis. J. Crohns Colitis 8, 1569–1581. doi: 10.1016/j.crohns.2014.08.006 25223604 PMC4296742

[B26] Cox L. M. Weiner H. L. (2018). Microbiota Signaling Pathways That Influence Neurologic Disease. Neurotherapeutics 15, 135–145. doi: 10.1007/s13311-017-0598-8 29340928 PMC5794708

[B27] Dabour N. Zihler A. Kheadr E. Lacroix C. Fliss I. (2009). *In Vivo* Study on the Effectiveness of Pediocin PA-1 and *Pediococcus Acidilactici* UL5 at Inhibiting *Listeria Monocytogenes* . Int. J. Food Microbiol. 133, 225–233. doi: 10.1016/j.ijfoodmicro.2009.05.005 19541383

[B28] D’amelio P. Sassi F. (2018). Gut Microbiota, Immune System, and Bone. Calcif. Tissue Int. 102, 415–425. doi: 10.1007/s00223-017-0331-y 28965190

[B29] De Kwaadsteniet M. Doeschate K. T. Dicks L. M. T. (2009). Nisin F in the Treatment of Respiratory Tract Infections Caused by *Staphylococcus Aureus.* Lett. Appl. Microbiol. 48, 65–70. doi: 10.1111/j.1472-765X.2008.02488.x 19018962

[B30] De Kwaadsteniet M. Fraser T. Van Reenen C. A. Dicks L. (2006). Bacteriocin T8, a Novel Class IIa Sec-Dependent Bacteriocin Produced by *Enterococcus Faecium* T8, Isolated From Vaginal Secretions of Children Infected With Human Immunodeficiency Virus. Appl. Environ. Microbiol. 72, 4761–4766. doi: 10.1128/AEM.00436-06 16820469 PMC1489345

[B31] Delcenserie V. Martel D. Lamoureux M. Amiot J. Boutin Y. Roy D. (2008). Immunomodulatory Effects of Probiotics in the Intestinal Tract. Curr. Issues Mol. Biol. 10, 37–54. doi: 10.21775/cimb.010.037 18525105

[B32] Dey D. Ema T. I. Biswas P. Aktar S. Islam S. Rinik U. R. (2021). Antiviral Effects of Bacteriocin Against Animal-to-Human Transmittable Mutated Sars-Cov-2: A Systematic Review. Front. Agr. Sci. Eng. 8, 603–622. doi: 10.15302/J-FASE-2021397

[B33] Dinan T. G. Cryan J. F. (2017). The Microbiome-Gut-Brain Axis in Health and Disease. Gastroenterol. Clin. North Am. 46, 77–89. doi: 10.1016/j.gtc.2016.09.007 28164854

[B34] Epand R. M. Walker C. Epand R. F. Magarvey N. A. (2016). Molecular Mechanisms of Membrane Targeting Antibiotics. Biochim. Biophys. Acta – Biomembr. 1858, 980–987. doi: 10.1016/j.bbamem.2015.10.018 26514603

[B35] Foligne B. Zoumpopoulou G. Dewulf J. Ben Younes A. Chareyre F. Sirard J. C. (2007). A Key Role of Dendritic Cells in Probiotic Functionality. PloSone 2, e313. doi: 10.1371/journal.pone.0000313 PMC1819555 17375199

[B36] Fong W. Li Q. Yu J. (2020). Gut Microbiota Modulation: A Novel Strategy for Prevention and Treatment of Colorectal Cancer. Oncogene 39, 4925–4943. doi: 10.1038/s41388-020-1341-1 32514151 PMC7314664

[B37] Furrie E. Macfarlane S. Kennedy A. Cummings J. H. Walsh S. V. O’neil D. A.. (2005). Synbiotic Therapy (*Bifidobacterium Longum*/Synergy 1) Initiates Resolution of Inflammation in Patients With Active Ulcerative Colitis: A Randomised Controlled Pilot Trial. Gut 54, 242–249. doi: 10.1136/gut.2004.044834 15647189 PMC1774839

[B38] Galland L. (2014). The Gut Microbiome and the Brain. J. Med. Food 17, 1261–1272. doi: 10.1089/jmf.2014.7000 25402818 PMC4259177

[B39] Gibson D. Mehler P. S. (2019). Anorexia Nervosa and the Immune System—a Narrative Review. J. Clin. Med. 8, 1–19. doi: 10.3390/jcm8111915 PMC6912362 31717370

[B40] Gillor O. Etzion A. Riley M. A. (2008). The Dual Role of Bacteriocins as Anti-and Probiotics. Appl. Microbiol. Biotechnol. 81, 591–606. doi: 10.1007/s00253-008-1726-5 18853155 PMC2670069

[B41] Goldstein B. P. Wei J. Greenberg K. Novick R. (1998). Activity of Nisin Against *Streptococcus Pneumoniae*, *In Vitro*, and in a Mouse Infection Model. J. Antimicrob. Chemother. 42, 277–278. doi: 10.1093/jac/42.2.277 9738856

[B42] Goyal C. Malik R. K. Pradhan D. (2018). Purification and Characterization of a Broad Spectrum Bacteriocin Produced by a Selected *Lactococcus Lactis* Strain 63 Isolated From Indian Dairy Products. J. Food Sci. Technol. 55, 3683–3692. doi: 10.1007/s13197-018-3298-4 30150828 PMC6098757

[B43] Harris L. J. Daeschel M. A. Stiles M. E. Klaenhammer T. R. (1989). Antimicrobial Activity of Lactic Acid Bacteria Against *Listeria Monocytogenes* . J. Food Prot. 52, 384–387. doi: 10.4315/0362-028X-52.6.384 31003307

[B44] Heeney D. D. Zhai Z. Bendiks Z. Barouei J. Martinic A. Slupsky C. (2019). Lactobacillus Plantarum Bacteriocin Is Associated With Intestinal and Systemic Improvements in Diet-Induced Obese Mice and Maintains Epithelial Barrier Integrity *In Vitro* . Gut Microbes 10, 382–397. doi: 10.1080/19490976.2018.1534513 30409105 PMC6546331

[B45] Heilbronner S. Krismer B. Brötz-Oesterhelt H. Peschel A. (2021). The Microbiome-Shaping Roles of Bacteriocins. Nat. Rev. Microbiol. 19, 726–739. doi: 10.1038/s41579-021-00569-w 34075213

[B46] Hernandez-Gonzalez J. C. Martínez-Tapia A. Lazcano-Hernández G. García-Pérez B. E. Castrejón-Jiménez N. S. (2021). Bacteriocins From Lactic Acid Bacteria. A Powerful Alternative as Antimicrobials, Probiotics, and Immunomodulators in Veterinary Medicine. Animals 11, 1–17. doi: 10.3390/ani11040979 PMC8067144 33915717

[B47] Hoarau C. Lagaraine C. Martin L. Velge-Roussel F. Lebranchu Y. (2006). Supernatant of *Bifidobacterium Breve* Induces Dendritic Cell Maturation, Activation, and Survival Through a Toll-Like Receptor 2 Pathway. J. Allergy Clin. Immunol. Pract. 117, 696–702. doi: 10.1016/j.jaci.2005.10.043 16522473

[B48] Huang F. Teng K. Liu Y. Cao Y. Wang T. Ma C (2021). Bacteriocins: Potential for Human Health. Oxid. Med. Cell. Longevity 212, 1–17. doi: 10.1155/2021/5518825 PMC8055394 33936381

[B49] Ilinskaya O. N. Ulyanova V. V. Yarullina D. R. Gataullin I. G. (2017). Secretome of Intestinal Bacilli: A Natural Guard Against Pathologies. Front. Microbiol. 8, 1666. doi: 10.3389/fmicb.2017.01666 28919884 PMC5586196

[B50] Jia Z. Chen A. Bao F. He M. Gao S. Xu J. (2018). Effect of Nisin on Microbiome-Brain-Gut Axis Neurochemicals by *Escherichia Coli*-Induced Diarrhea in Mice. Microb. Pathog. 119, 65–71. doi: 10.1016/j.micpath.2018.04.005 29649517

[B51] Johnson K. V. A. Foster K. R. (2018). Why Does the Microbiome Affect Behaviour? Nat. Rev. Microbiol. 16, 647–655. doi: 10.1038/s41579-018-0014-3 29691482

[B52] Kaur S. Kaur S. (2015). Bacteriocins as Potential Anticancer Agents. Front. Pharmacol. 6, 272. doi: 10.3389/fphar.2015.00272 26617524 PMC4639596

[B53] Kim S. G. Becattini S. Moody T. U. Shliaha P. V. Littmann E. R. Seok R. (2019). Microbiota-Derived Lantibiotic Restores Resistance Against Vancomycin-Resistant *Enterococcus* . Nature 572, 665–669. doi: 10.1038/s41586-019-1501-z 31435014 PMC6717508

[B54] Kling A. Lukat P. Almeida D. V. Bauer A. Fontaine E. Sordello S. (2015). Targeting DnaN for Tuberculosis Therapy Using Novel Griselimycins. Science 348, 1106–1112. doi: 10.1126/science.aaa4690 26045430

[B55] Krebs B. (2016). Prebiotic and Synbiotic Treatment Before Colorectal Surgery-Randomised Double Blind Trial. Coll. Antropol. 40, 35–40. 27301235

[B56] Kwok L. Y. Guo Z. Zhang J. Wang L. Qiao J. Hou Q. (2015). The Impact of Oral Consumption of *Lactobacillus Plantarum* P-8 on Faecal Bacteria Revealed by Pyrosequencing. Benef Microbes 64, 05–413. doi: 10.3920/BM2014.0063 25653153

[B57] Lange K. Buerger M. Stallmach A. Bruns T. (2016). Effects of Antibiotics on Gut Microbiota. Dig. Dis. Sci. 34, 260–268. doi: 10.1159/000443360 27028893

[B58] Lange-Starke A. Petereit A. Truyen U. Braun P. G. Fehlhaber K. Albert T. (2014). Antiviral Potential of Selected Starter Cultures, Bacteriocins and D, L-Lactic Acid. Food Environ. Virol. 6, 42–47. doi: 10.1007/s12560-013-9135-z 24297091 PMC7090810

[B59] Lehtoranta L. Latvala S. Lehtinen M. J. (2020). Role of Probiotics in Stimulating the Immune System in Viral Respiratory Tract Infections: A Narrative Review. Nutrients 12, 3163. doi: 10.3390/nu12103163 PMC7602805 33081138

[B60] Liou A. P. Paziuk M. Luevano J. M. Machineni S. Turnbaugh P. J. Kaplan L. M. (2013). Conserved Shifts in the Gut Microbiota Due to Gastric Bypass Reduce Host Weight and Adiposity. Sci. Transl. Med. 5, 178ra41–178ra41. doi: 10.1126/scitranslmed.3005687 PMC3652229 23536013

[B61] Liu G. Ren W. Fang J. Hu C. A. A. Guan G. Al-Dhabi N. A. (2017). L-Glutamine and L-Arginine Protect Against Enterotoxigenic *Escherichia Coli* Infection *via* Intestinal Innate Immunity in Mice. Amino Acids 49, 1945–1954. doi: 10.1007/s00726-017-2410-9 28299479

[B62] Liu Q. Yu Z. Tian F. Zhao J. Zhang H. Zhai Q. (2020). Surface Components and Metabolites of Probiotics for Regulation of Intestinal Epithelial Barrier. Microb. Cell Factories 19, 1–11. doi: 10.1186/s12934-020-1289-4 PMC7003451 32024520

[B63] Li Y. Xia S. Jiang X. Feng C. Gong S. Ma J. (2021). Gut Microbiota and Diarrhea: An Updated Review. Front. Cell. Infect. Microbiol. 11, 625210. doi: 10.3389/fcimb.2021.625210 33937093 PMC8082445

[B64] Li Q. Yu S. Han J. Wu J. You L. Shi X. (2022). Synergistic Antibacterial Activity and Mechanism of Action of Nisin/Carvacrol Combination Against Staphylococcus Aureus and Their Application in the Infecting Pasteurized Milk. Food Chem. 380, 132009. doi: 10.1016/j.foodchem.2021.132009 35077986

[B65] Macpherson A. J. Geuking M. B. McCoy K. D. (2005). Immune Responses That Adapt the Intestinal Mucosa to Commensal Intestinal Bacteria. Immunology 115, 153–162. doi: 10.1111/j.1365-2567.2005.02159.x 15885120 PMC1782138

[B66] Macpherson A. J. Uhr T. (2004). Induction of Protective IgA by Intestinal Dendritic Cells Carrying Commensal Bacteria. Sci. (New York N.Y.) 303, 1662–1665. doi: 10.1126/science.1091334 15016999

[B67] Martin-Visscher L. A. van Belkum M. J. Garneau-Tsodikova S. Whittal R. M. Zheng J. McMullen L. M. (2008). Isolation and Characterization of Carnocyclin A, a Novel Circular Bacteriocin Produced by *Carnobacterium Maltaromaticum* UAL307. Appl. Environ. Microbiol. 74, 4756–4763. doi: 10.1128/AEM.00817-08 18552180 PMC2519327

[B68] Mazloom K. Siddiqi I. Covasa M. (2019). Probiotics: How Effective Are They in the Fight Against Obesity? Nutrients 11, 258. doi: 10.3390/nu11020258 PMC6412733 30678355

[B69] Mazmanian S. K. Mazamaniam S. Round J. L. Kasper D. L. (2008). A Microbial Symbiosis Factor Prevents Intestinal Inflammatory Disease. Nature 453, 620–625. doi: 10.1038/nature07008 18509436

[B70] Michielan A. D’Incà R. (2015). Intestinal Permeability in Inflammatory Bowel Disease: Pathogenesis, Clinical Evaluation, and Therapy of Leaky Gut. Mediators Inflamm. 2015, 1–10. doi: 10.1155/2015/628157 PMC4637104 26582965

[B71] Mileti E. Matteoli G. Iliev I. D. Rescigno M. (2009). Comparison of the Immunomodulatory Properties of Three Probiotic Strains of Lactobacilli Using Complex Culture Systems: Prediction for *In Vivo* Efficacy. PloSone 4, e7056. doi: 10.1371/journal.pone.0007056 PMC2738944 19756155

[B72] Millette M. Cornut G. Dupont C. Shareck F. Archambault D. Lacroix M. (2008). Capacity of Human Nisin and Pediocin-Producing Lactic Acid Bacteria to Reduce Intestinal Colonization by Vancomycin-Resistant Enterococci. Appl. Environ. Microbiol. 74, 1997–2003. doi: 10.1128/AEM.02150-07 18245231 PMC2292579

[B73] Million M. Lagier J. C. Yahav D. Paul M. (2013). Gut Bacterial Microbiota and Obesity. Clin. Microbiol. Infect. 19, 305–313. doi: 10.1111/1469-0691.12172 23452229

[B74] Mokoena M. P. (2017). Lactic Acid Bacteria and Their Bacteriocins: Classification, Biosynthesis and Applications Against Uropathogens: A Mini-Review. Molecules 22, 1255. doi: 10.3390/molecules22081255 PMC6152299 28933759

[B75] Nagpal R. Mainali R. Ahmadi S. Wang S. Singh R. Kavanagh K. (2018). Gut Microbiome and Aging: Physiological and Mechanistic Insights. J. Nutr. Health Aging 4, 267–285. doi: 10.3233/NHA-170030 PMC6004897 29951588

[B76] Negash A. W. Tsehai B. A. (2020). Current Applications of Bacteriocin. Int. J. Microbiol. 2020, 1–7. doi: 10.1155/2020/4374891 PMC7803181 33488719

[B77] Nishei M. Nagao J. Sonomoto K. (2012). Antibacterial Peptide Bacteriocin. An Overview of Their Diverse Characteristics and Applications. Biocontrol Sci. 1, 1–16. doi: 10.4265/bio.17.1 22451427

[B78] Norouzi Z. Salimi A. Halabian R. Fahimi H. (2018). Nisin, a Potent Bacteriocin and Anti-Bacterial Peptide, Attenuates Expression of Metastatic Genes in Colorectal Cancer Cell Lines. Microb. Pathog. 123, 183–189. doi: 10.1016/j.micpath.2018.07.006 30017942

[B79] O’Callaghan A. Sinderen D. (2016). Bifidobacteria and Their Role as Members of the Human Gut Microbiota. Front. Microbiol. 7, 925. doi: 10.3389/fmicb.2016.00925 27379055 PMC4908950

[B80] O’ Connor P. M. O’ Shea E. F. Cotter P. D. Hill C. Ross R. P. (2018). The Potency of the Broad Spectrum Bacteriocin, Bactofencin A, Against Staphylococci Is Highly Dependent on Primary Structure, N-Terminal Charge and Disulphide Formation. Sci. Rep. 8, 1–8. doi: 10.1038/s41598-018-30271-6 30087409 PMC6081437

[B81] Pablo M. A. Gaforio J. J. Gallego A. M. Ortega E. Gálvez A. M. Alvarez de Cienfuegos López G. (1999). Evaluation of Immunomodulatory Effects of Nisin-Containing Diets on Mice. FEMS Microbiol. Immunol. 24, 35–42. doi: 10.1111/j.1574-695X.1999.tb01262.x 10340710

[B82] Papaconstantinou H. T. Thomas J. S. (2007). Bacterial Colitis. Clin. Colon Rectal Surg. 20, 018–027. doi: 10.1055/s-2007-970196 PMC2780149 20011357

[B83] Perez R. H. Zendo T. Sonomoto K. (2014). Novel Bacteriocins From Lactic Acid Bacteria (LAB): Various Structures and Applications. Microb. Cell Fact 13, S3. doi: 10.1186/1475-2859-13-S1-S3 25186038 PMC4155820

[B84] Pérez-Ramos A. Madi-Moussa D. Coucheney F. Drider D. (2021). Current Knowledge of the Mode of Action and Immunity Mechanisms of LAB-Bacteriocins. Microorganisms 9 (10), 2107. doi: 10.3390/microorganisms9102107 34683428 PMC8538875

[B85] Preet S. Bharati S. Panjeta A. Tewari R. Rishi P. (2015). Effect of Nisin and Doxorubicin on DMBA-Induced Skin Carcinogenesis—a Possible Adjunct Therapy. Tumor Biol. 36, 8301–8308. doi: 10.1007/s13277-015-3571-3 26002579

[B86] Quigley E. M. (2017). Microbiota-Brain-Gut Axis and Neurodegenerative Diseases. Curr. Neurol. Neurosci. Rep. 17, 1–9. doi: 10.1007/s11910-017-0802-6 29039142

[B87] Rea M. C. Clayton E. O’Connor P. M. Shanahan F. Kiely B. Ross R. P. (2007). Antimicrobial Activity of Lacticin 3147 Against Clinical *Clostridium Difficile* Strains. J. Med. Microbiol. 56, 940– 946. doi: 10.1099/jmm.0.47085-0 17577060

[B88] Rea M. C. Sit C. S. Clayton E. O’Connor P. M. Whittal R. M. Zheng J. (2010). Thuricin CD, a Posttranslationally Modified Bacteriocin With a Narrow Spectrum of Activity Against *Clostridium Difficile* . Proc. Natl. Acad. Sci. 107, 9352–9357. doi: 10.1073/pnas.0913554107 20435915 PMC2889069

[B89] Riboulet-Bisson E. Sturme M. H. J. Jeffery I. B. O’Donnell M. M. Neville B. A. Forde B. M. (2012). Effect of *Lactobacillus Salivarius* Bacteriocin Abp118 on the Mouse and Pig Intestinal Microbiota. PloS One 7, 1–12. doi: 10.1371/journal.pone.0031113 PMC3281923 22363561

[B90] Ryma T. Samer A. Soufli I. Rafa H. Touil-Boukoffa C. (2021). Role of Probiotics and Their Metabolites in Inflammatory Bowel Diseases (IBDs). Gastroenterol. Insights 12, 56–66. doi: 10.3390/gastroent12010006

[B91] Salman J. A. S. Mahmood N. N. Abdulsattar B. O. Abid H. A. (2020). The Effectiveness of Probiotics Against Viral Infections: A Rapid Review With Focus on SARS-CoV-2 Infection. Open Access Maced. J. Med. Sci. 8, 496–508. doi: 10.3889/oamjms.2020.5483

[B92] Salvucci E. Saavedra L. Hebert E. Haro C. Sesma F. (2012). Enterocin CRL35 Inhibits *Listeria Monocytogenes* in a Murine Model. Foodborne Pathog. Dis. 9, 68–74. doi: 10.1089/fpd.2011.0972 22011041

[B93] Sanders M. E. Heimbach J. T. Pot B. Tancredi D. J. Lenoir-Wijnkoop I. Lähteenmäki-Uutela A. (2011). Health Claims Substantiation for Probiotic and Prebiotic Products. Gut Microbes 2, 127–133. doi: 10.4161/gmic.2.3.16174 21646865

[B94] Sassone-Corsi M. Nuccio S. P. Liu H. Hernandez D. Vu C. T. Takahashi A. A. (2016). Microcins Mediate Competition Among Enterobacteriaceae in the Inflamed Gut. Nature 540, 280–283. doi: 10.1038/nature20557 27798599 PMC5145735

[B95] Saunders P. R. Santos J. Hanssen N. P. Yates D. Groot J. A. Perdue M. H. (2002). Physical and Psychological Stress in Rats Enhances Colonic Epithelial Permeability *via* Peripheral CRH. Dig. Dis. Sci. 47, 208–215. doi: 10.1023/A:1013204612762 11852879

[B96] Scaldaferri F. Gerardi V. Lopetuso L. R. Del Zompo F. Mangiola F. Boškoski I. (2013). Gut Microbial Flora, Prebiotics, and Probiotics in IBD: Their Current Usage and Utility. Biomed. Res. Int. 2010, 1–10. doi: 10.1155/2013/435268 PMC3749555 23991417

[B97] Schlee M. Miriam S. Wehkamp J. Altenhoefer A. Oelschlaeger T. A. Stange E. F.. (2007). Induction of Human β-Defensin 2 by the Probiotic Escherichia Coli Nissle 1917 Is Mediated Through Flagellin. Infect. Immun. 75, 2399–2407. doi: 10.1128/IAI.01563-06 17283097 PMC1865783

[B98] Shadnoush M. Hosseini R. S. Khalilnezhad A. Navai L. Goudarzi H. Vaezjalali M. (2015). Effects of Probiotics on Gut Microbiota in Patients With Inflammatory Bowel Disease: A Double-Blind, Placebo-Controlled Clinical Trial. Korean J. Gastroenterol. 65, 215–221. doi: 10.4166/kjg.2015.65.4.215 25896155

[B99] Sheoran P. Tiwari S. K. (2021). Synergistically-Acting Enterocin LD3 and Plantaricin LD4 Against Gram-Positive and Gram-Negative Pathogenic Bacteria. Probiotics Antimicro. Prot. 13, 542–554. doi: 10.1007/s12602-020-09708-w PMC7486809 32918678

[B100] Shin J. M. Gwak J. W. Kamarajan P. Fenno J. C. Rickard A. H. Kapila Y. L. (2016). Biomedical Applications of Nisin. J. Appl. Microbiol. 120, 1449–1465. doi: 10.1111/jam.13033 26678028 PMC4866897

[B101] Sidhu M. Vander Poorten D. (2017). The Gut Microbiome. Aust. Fam. Physician 46, 206–211. 28376573

[B102] Singh K. Rao A. (2021). Probiotics: A Potential Immunomodulator in COVID-19 Infection Management. Nutr. Res. 87, 1–12. doi: 10.1016/j.nutres.2020.12.014 33592454 PMC7881295

[B103] Soleimanpour S. Hasanian S. M. Avan A. Yaghoubi A. Khazaei M. (2020). Bacteriotherapy in Gastrointestinal Cancer. Life Sci. 254, 117754. doi: 10.1016/j.lfs.2020.117754 32389833

[B104] Soltani S. Hammami R. Cotter P. D. Rebuffat S. Said L. B. Gaudreau H. (2021). Bacteriocins as a New Generation of Antimicrobials: Toxicity Aspects and Regulations. FEMS Microbiol. Rev. 45, 39. doi: 10.1093/femsre/fuaa039 PMC7794045 32876664

[B105] Sze M. A. Schloss P. D. (2016). Looking for a Signal in the Noise: Revisiting Obesity and the Microbiome. MBio 7, 1018. doi: 10.1128/mBio.01018-16 PMC4999546 27555308

[B106] Taherikalani M. Ghafourian S. (2021). Anticancer Properties of Colicin E7 Against Colon Cancer. Prz. Gastroenterol. 16, 364. doi: 10.5114/pg.2021.109622 34976246 PMC8690953

[B107] Tiwari S. K. Dicks L. M. Popov I. V. Karaseva A. Ermakov A. M. Suvorov A. (2020). Probiotics at War Against Viruses: What Is Missing From the Picture? Front. Microbiol. 11, 1877. doi: 10.3389/fmicb.2020.01877 32973697 PMC7468459

[B108] Todorov S. D. Wachsman M. Tomé E. Dousset X. Destro M. T. Dicks L. M. T. (2010). Characterisation of an Antiviral Pediocin-Like Bacteriocin Produced by *Enterococcus Faecium* . Food Microbiol. 27, 869–879. doi: 10.1016/j.fm.2010.05.001 20688228

[B109] Turnbaugh P. J. Bäckhed F. Fulton L. Gordon J. I. (2008). Diet-Induced Obesity Is Linked to Marked But Reversible Alterations in the Mouse Distal Gut Microbiome. Cell Host Microbe 3, 213–223. doi: 10.1016/j.chom.2008.02.015 18407065 PMC3687783

[B110] Umu Ö.C. Bäuerl C. Oostindjer M. Pope P. B. Hernández P. E. Pérez-Martínez G. (2016). The Potential of Class II Bacteriocins to Modify Gut Microbiota to Improve Host Health. PloS One 11, 1–22. doi: 10.1371/journal.pone.0164036 PMC5047636 27695121

[B111] Umu Ö.C. Rudi K. Diep D. B. (2017). Modulation of the Gut Microbiota by Prebiotic Fibres and Bacteriocins. Microb. Ecol. Health Dis. 28, 1–12. doi: 10.1080/16512235.2017.1348886 PMC5614387 28959178

[B112] Van-Belkum M. J. Martin-Visscher L. A. Vederas J. C. (2011). Structure and Genetics of Circular Bacteriocins. Trends Microbiol. 19, 411–418. doi: 10.1016/j.tim.2011.04.004 21664137

[B113] Villena J. Shimosato T. Vizoso-Pinto M. G. Kitazawa H. (2020). Nutrition, Immunity and Viral Infections. Front. Nutr. 7, 125. doi: 10.3389/fnut.2020.00125 PMC7507487 33015114

[B114] Wachsman M. B. Farıías M. E. Takeda E. Sesma F. De Ruiz Holgado A. P. De Torres R. A. (1999). Antiviral Activity of Enterocin CRL35 Against Herpesviruses. Int. J. Antimicrob. Agents 12, 293–299. doi: 10.1016/S0924-8579(99)00078-3 10493605

[B115] Walter J. (2008). Ecological Role of Lactobacilli in the Gastrointestinal Tract: Implications for Fundamental and Biomedical Research. Appl. Environ. Microbiol. 74, 4985–4996. doi: 10.1128/AEM.00753-08 18539818 PMC2519286

[B116] Wang H. X. Wang Y. P. (2016). Gut Microbiota-Brain Axis. Chin. Med. J. 129, 2373–2380. doi: 10.4103/0366-6999.190667 27647198 PMC5040025

[B117] Wiciński M. Gębalski J. Gołębiewski J. Malinowski B. (2020). Probiotics for the Treatment of Overweight and Obesity in Humans—A Review of Clinical Trials. Microorganisms 8, 1148. doi: 10.3390/microorganisms8081148 PMC7465252 32751306

[B118] Wu S. Yuan L. Zhang Y. Liu F. Li G. Wen K. (2013). Probiotic Lactobacillus Rhamnosus GG Mono-Association Suppresses Human Rotavirus-Induced Autophagy in the Gnotobiotic Piglet Intestine. Gut Pathog. 5, 22. doi: 10.1186/1757-4749-5-22 23924832 PMC3750464

[B119] Xue L. He J. Gao N. Lu X. Li M. Wu X. (2017). Probiotics may Delay the Progression of Nonalcoholic Fatty Liver Disease by Restoring the Gut Microbiota Structure and Improving Intestinal Endotoxemia. Sci. Rep. 7, 1–13. doi: 10.1038/srep45176 28349964 PMC5368635

[B120] Yang S. C. Lin C. H. Sung C. T. Fang J. Y. (2014). Antibacterial Activities of Bacteriocins: Application in Foods and Pharmaceuticals. Front. Microbiol. 5, 241. doi: 10.3389/fmicb.2014.00241 24904554 PMC4033612

[B121] Yan H. Lu Y. Li X. Yi Y. Wang X. Shan Y (2021). Action Mode of Bacteriocin BM1829 Against *Escherichia Coli* and *Staphylococcus Aureus* . Food Biosci. 39, 100794. doi: 10.1016/j.fbio.2020.100794

[B122] Yun B. Oh S. Griffiths M. W. (2014). Lactobacillus Acidophilus Modulates the Virulence of *Clostridium Difficile* . J. Dairy Sci. 97, 4745–4758. doi: 10.3168/jds.2014-7921 24856984

[B123] Zacharof M. P. Lovitt R. W. (2012). Bacteriocins Produced by Lactic Acid Bacteria a Review Article. Apcbee Proc. 2, 50–56. doi: 10.1016/j.apcbee.2012.06.010

[B124] Zeuthen L. H. Fink L. N. Frøkiaer H. (2008). Toll-Like Receptor 2 and Nucleotide Binding Oligomerization Domain-2 Play Divergent Roles in the Recognition of Gut-Derived Lactobacilli and Bifidobacteria in Dendritic Cells. Immunology 124, 489–502. doi: 10.1111/j.1365-2567.2007.02800.x 18217947 PMC2492941

[B125] Zhang Y.-J. Li S. Gan R.-Y. Zhou T. Xu D.-P. Li H.-B. (2015). Impacts of Gut Bacteria on Human Health and Disease. Int. J. Mol. Sci. 16, 7493–7519. doi: 10.3390/ijms16047493 25849657 PMC4425030

[B126] Zheng J. Wittouck S. Salvetti E. Franz C. M. Harris H. Mattarelli P. (2020). A Taxonomic Note on the Genus Lactobacillus: Description of 23 Novel Genera, Emended Description of the Genus Lactobacillus Beijerinck 1901, and Union of Lactobacillaceae and Leuconostocaceae. Int. J. Syst. Evol. Microbiol. 70, 2782–2858. doi: 10.7939/r3-egnz-m294 32293557

[B127] Zhong L. Zhang X. Covasa M. (2014). Emerging Roles of Lactic Acid Bacteria in Protection Against Colorectal Cancer. World J. Gastroenterol. 20, 7878–7886. doi: 10.3748/wjg.v20.i24.7878 24976724 PMC4069315

